# Isolation and characterization of 11 novel microsatellite loci in a West African leaf-nosed bat, *Hipposideros* aff. *ruber*

**DOI:** 10.1186/1756-0500-7-607

**Published:** 2014-09-04

**Authors:** Heather J Baldwin, Peter Vallo, Michael G Gardner, Christian Drosten, Marco Tschapka, Adam J Stow

**Affiliations:** Institute of Experimental Ecology, University of Ulm, Albert-Einstein-Allee 11, 89069 Ulm, Germany; Department of Biological Sciences, Macquarie University, Sydney, New South Wales 2109 Australia; Institute of Vertebrate Biology, v. v. i., Academy of Sciences of the Czech Republic, Květná 8, 603 65 Brno, Czech Republic; School of Biological Sciences, Flinders University, Adelaide, South Australia 5001 Australia; Evolutionary Biology Unit, South Australian Museum, North Terrace, Adelaide, South Australia 5000 Australia; Institute of Virology, University of Bonn Medical Center, Sigmund-Freud-Str 25, 53105 Bonn, Germany; Smithsonian Tropical Research Institute, PO Box 0843–03092, Balboa, Panama

**Keywords:** *Hipposideros ruber*, *Hipposideros caffer*, Microsatellites, Population genetics, Microchiroptera, Hipposideridae, Bat

## Abstract

**Background:**

Noack’s leaf-nosed bat, *Hipposideros ruber*, is a cryptic species within the *Hipposideros caffer* species complex. Despite a widespread distribution in Africa and being host to potentially zoonotic viruses, the genetic structure and ecology of *H. ruber* is poorly known. Here we describe the development of 11 novel polymorphic microsatellite loci to facilitate the investigation of genetic structure.

**Findings:**

We selected 20 microsatellite sequences identified from high throughput sequence reads and PCR amplified these for 38 individuals, yielding 11 consistently amplifying and scorable loci. The number of alleles per locus ranged from two to 12, and observed heterozygosities from 0.00 to 0.865. No evidence of linkage disequilibrium was observed, and nine of the markers showed no departure from Hardy-Weinberg equilibrium. We demonstrate successful amplification in two closely related species and two divergent lineages of the *H. caffer* species complex.

**Conclusions:**

These new markers will provide a valuable tool to investigate genetic structure in the poorly understood *Hipposideros caffer* species complex.

## Findings

Noack’s leaf-nosed bat *Hipposideros ruber* (Noack, 1893) is one of two recognised cryptic species within the *Hipposideros caffer* (Sundevall, 1846) species complex. These bats are widespread throughout sub-Saharan Africa and among the most abundant mammals on the continent [1, 2]. Mitochondrial evidence has shown the existence of several deeply divergent lineages within the *H. caffer* complex, which most likely constitute more than the two species [3]. Recently, they have been discovered to host viruses with zoonotic potential [4], emphasizing the need for knowledge about their ecology in order to gain insight into zoonotic processes and risk factors for public health. Microsatellites provide a powerful tool to investigate the poorly known ecology and life history of these bats, including genetic structure, social arrangements and mating systems. Assessment of nuclear gene flow through microsatellite analysis may thus help to shed light also on the taxonomy of this species complex.

We isolated and characterized 11 microsatellite loci from a single, exclusively West African mitochondrial lineage of *Hipposideros ruber*, determined by sequencing of the cytochrome *b* gene [lineage D; 3]. This lineage is henceforth called *H.* aff. *ruber* due to its distant evolutionary relationship to *H. ruber* s. str. from East Africa, and may represent a distinct species [3]. *Hipposideros* aff. *ruber* has been previously identified in central Ghana [5] and seems to be the most abundant of the three main lineages of the *H. caffer* complex in this region (unpublished data). The markers described herein represent, to our knowledge, the first suite of microsatellites for an African hipposiderid bat.

DNA was extracted from wing tissue from eight individuals sampled from the Brong Ahafo and Volta regions in central Ghana. DNA was extracted using an innuPREP DNA mini kit (Analytik Jena, Jena, Germany). Five micrograms of pooled DNA from eight individuals was sent to AGRF (http://www.agrf.com.au), where high throughput sequencing was performed on a Roche GS FLX 454 sequencing machine as described elsewhere [6–8]. QDD 1.3 [9] was used to screen for di- to hexanucleotide repeat motifs with a minimum of eight repeats. From the 1689 microsatellites identified, a total of 32 primer pairs flanking tetranucleotide repeats with 11–15 repeat motifs were designed using PRIMER3 [10]. Twenty primer pairs for which the annealing temperatures were most similar for each primer were selected for initial amplification trials. Amplification products from these primer pairs were visualised by electrophoresis on an agarose gel, from which 13 pairs with strong, stutter-free amplification bands were selected for optimisation. Forward primers for these 13 loci were directly labelled with a fluorochrome at the 5′ end. Twelve of these loci were successfully amplified by polymerase chain reaction (PCR), with one discarded due to the excessive amplification of non-specific product. PCR conditions for these 12 loci were optimized and genotyping was performed on 38 individuals (16 females, 22 males) sampled in Brong Ahafo and Volta Regions.

PCRs were performed using 10–50 ng of template DNA and reagent concentrations as follows: 200 μM each dNTP, one unit reaction buffer, between 2.0 and 2.5 mM MgCl_2_, equal concentrations of forward and reverse primer (0.25–1.0 μM) and one unit *Taq* polymerase (see Table 1). PCR amplification consisted of an initial denaturation at 94°C for 3 min followed by six touchdown cycles of 94°C denaturation for 30 s, annealing for 30 s with temperatures decreased by 2°C per cycle (55–47°C, 60–50°C, or 65–55°C; Table 1), and polymerase extension step at 72°C for 45 s. An additional 35 cycles were conducted, of denaturation (94°C, 30 s), primer annealing (final touchdown temperature, 45 s), and polymerase extension (72°C, 45 s), followed by a final extension (72°C, 10 min). PCR products were electrophoresed using an ABI3130 Genetic Analyzer (Applied Biosystems, Foster City, CA, USA). Allele sizes were determined via manual inspection using the software PEAK SCANNER 1.0 (Applied Biosystems), followed by automated binning performed using TANDEM 1.09 [11]. We reanalyzed 20% of individuals to evaluate data integrity. One locus (Hr3) was discarded due to high rounding error in the TANDEM analysis, indicating poor marker quality. MICRO-CHECKER 2.2.3 was used to assess the probability of scoring errors, allelic dropout and the presence of null alleles [12]. No scoring errors or allelic dropout were detected, although there were potentially null alleles at loci Hr7 and Hr12. Locus Hr13 may also suffer from null alleles, though low allelic variability (one common and one rare allele) did not allow this to be confirmed (Table 1).

**Table 1:** **Characteristics and thermocycling conditions for 11 polymorphic microsatellites in the African leaf-nosed bat** ***Hipposideros*** **aff.** ***ruber***

Locus	Accession #	Repeat motif	Primer sequences (5′ – 3′)	MgCl _2_/***P***_FR_	***T***a (°C)	Size (bp)	N	N _A_	H _O_	H _E_	HWE	P _NULL_
**Hr1**	KM370156	(GATA)_13_	F:TGGCAAGGTTAACACGAACC	2.0 mM/0.5 μM	60-50	238-258	38	6	0.74	0.79	ns	0.028
			R:TCTCCCTCCCGCTCTTATCT									
**Hr2**	KM370157	(TCTT)_15_	F:GAAGCACTGCTGGAAAGGTT	2.0 mM/0.25 μM	60-50	311-339	34	8	0.77	0.76	ns	-0.009
			R:GTTGAACTGGGTGGCCTTTA									
**Hr5**	KM370160	(GAAG)_14_	F:TGGGTGTTTCAGTTTCATGC	2.0 mM/0.5 μM	65-55	186-234	34	9	0.82	0.82	ns	-0.006
			R:TGGTCTATTTGTTTCCTTCCGTA									
**Hr6**	KM370161	(TCTT)_13_	F:GGGTTTCTTCAAATGTGTTTTC	2.0 mM/0.5 μM	55-47	204-240	37	8	0.70	0.73	ns	0.012
			R:GCCTCCAAGACAAACAGAGG									
**Hr7**	KM370162	(ATTT)_11_	F:AGCCAATGACAAGACTGCCTA	2.0 mM/0.5 μM	65-55	144-172	33	8	0.42	0.68	***	0.173
			R:CCAGTGAAGCAACGTCCTCT									
**Hr8**	KM370163	(ATCT)_12_	F:CTCAGCCCAAAGTCAAGGAG	2.0 mM/0.5 μM	60-50	221-241	36	6	0.72	0.68	ns	-0.042
			R:TGGCTATACGAATACAAAGATTAGACA									
**Hr9**	KM370164	(TCTA)_12_	F:TGCTATCTTCCATGAGGTCAGA	2.0 mM/0.5 μM	60-50	218-234	38	5	0.63	0.73	ns	0.061
			R:TCTCTGTTGCTGAAGGAAAACTT									
**Hr10**	KM370165	(TTAT)_11_	F:TCCACTGGAGTAAGAGATGTGTG	2.0 mM/1.0 μM	65-55	258-282	38	7	0.79	0.74	ns	-0.040
			R:GCACTGCAACAGTGAAAAGC									
**Hr11**	KM370166	(TTTC)_14_	F:CTCTTGCAATGAAGGCAATG	2.0 mM/0.5 μM	65-55	106-154	37	12	0.87	0.86	ns	-0.018
			R:CTGCCATGAGCTACCATGAG									
**Hr12**	KM370167	(GATA)_12_	F:TTGGTTTTCAGATCTTCTGGTG	2.5 mM/0.5 μM	60-50	277-293	38	4	0.42	0.60	**	0.140
			R:GAGTCTTCTGCCTGCTGGAC									
**Hr13**	KM370168	(TTTC)_13_	F:CCGAAGCCAATCTGGTTTTA	2.0 mM/1.0 μM	65-55	321-329	34	2	0.00	0.06	ns	0.157
			R:GGGTCCTGCAGAAACACACT									

*P*_FR_ forward and reverse primer concentration, *T*a annealing temperatures of touchdown cycles (see Methods), N number of individuals, N_A_ number of alleles, H_O_ observed heterozygosity, H_E_ expected heterozygosity, HWE probability of deviation from Hardy-Weinberg equilibrium, P_NULL_ null allele frequency estimate (van Oosterhout), ns not significant., **p < 0.01, ***p < 0.001.

The program CERVUS was used to calculate number of alleles, observed (*H*_o_) and expected (*H*_e_) heterozygosities, and probabilities of identity [13]. All 11 loci were determined to be polymorphic, with a range of 2–12 alleles per locus (Table 1). Tests for pairwise linkage disequilibrium and deviations from Hardy-Weinberg equilibrium with Bonferroni corrections were calculated using FSTAT 2.9.3 [14]. Two loci (Hr7, Hr12) deviated significantly from the Hardy-Weinberg equilibrium with a homozygote excess (Table 1). No linkage disequilibrium was detected between any loci. The probability of identity for the 11 loci was low at 1.6E^-10^ overall, and 8.7E^-10^ and 3.1E^-9^ for the Brong Ahafo and Volta localities, respectively. Probability of sibling identity was 1.4E^-4^, 2.2E^-4^ and 2.8E^-4^ for overall, Brong Ahafo and Volta, respectively.

In order to explore utility in closely related taxa, we tested whether these loci could be amplified across four related taxa in the genus *Hipposideros* using the PCR conditions specified above (Table 2). All but one locus successfully amplified PCR product across the tested taxa.

**Table 2:** **Cross-amplification success in other** ***Hipposideros*** **species or lineages**

Taxon	Hr1	Hr2	Hr5	Hr6	Hr7	Hr8	Hr9	Hr10	Hr11	Hr12	Hr13
*H. abae*	+	+	+	+	+	+	+	+	+	+	+
*H. tephrus*	+	+	+	+	+	+	+	+	–	+	+
*H. ruber* (lin. B)^§^	+	+	+	+	+	+	+	+	+	+	+
*H. ruber* (lin. C)^§^	+	+	+	+	+	+	+	+	+	+	+

+successful amplification with 1–2 bands visualised of expected size, - no PCR product observed.

^§^sensu Vallo et al. 2008. lin. = lineage.

These microsatellite loci provide useful resources for the study of population genetic structure of bats in the *Hipposideros caffer* complex, and likely also related species in this genus. These findings will help to address questions regarding connectivity, social behaviour, and zoonotic disease ecology in African leaf-nosed bats.

### Ethics statement

All animals were handled in accordance with Ghanaian legislation. Bat capture and sampling were authorized by permit from the Wildlife Division of the Ministry of Lands, Forestry and Mines in Ghana, and approved by the Macquarie University Ethics Committee. Exports were conducted under a state agreement between the Republic of Ghana and the Federal Republic of Germany, and to Australia with permission from the Department of Agriculture, Fisheries and Forestry.

### Availability of the supporting data

The microsatellite sequences are available through the National Centre for Biotechnology Information (http://www.ncbi.nlm.nih.gov); GenBank accession numbers KM370156 – KM370168.
